# Wireless Micro Soft Actuator without Payloads Using 3D Helical Coils

**DOI:** 10.3390/mi13050799

**Published:** 2022-05-20

**Authors:** Seonghyeon Lee, Woojun Jung, Kyungho Ko, Yongha Hwang

**Affiliations:** Department of Control and Instrumentation Engineering, Korea University, Sejong 30019, Korea; kazamajin95@korea.ac.kr (S.L.); wjkst2010@korea.ac.kr (W.J.); gokyungho123@korea.ac.kr (K.K.)

**Keywords:** 3D helical inductors, wireless actuators, magnetic induction, liquid–gas phase changes, soft actuators

## Abstract

To receive a greater power and to demonstrate the soft bellows-shaped actuator’s wireless actuation, micro inductors were built for wireless power transfer and realized in a three-dimensional helical structure, which have previously been built in two-dimensional spiral structures. Although the three-dimensional helical inductor has the advantage of acquiring more magnetic flux linkage than the two-dimensional spiral inductor, the existing microfabrication technique produces a device on a two-dimensional plane, as it has a limit to building a complete three-dimensional structure. In this study, by using a three-dimensional printed soluble mold technique, a three-dimensional heater with helical coils, which have a larger heating area than a two-dimensional heater, was fabricated with three-dimensional receiving inductors for enhanced wireless power transfer. The three-dimensional heater connected to the three-dimensional helical inductor increased the temperature of the liquid and gas inside the bellows-shaped actuator while reaching 176.1% higher temperature than the heater connected to the two-dimensional spiral inductor. Thereby it enables a stroke of the actuator up to 522% longer than when it is connected to the spiral inductor. Therefore, three-dimensional micro coils can offer a significant approach to the development of wireless micro soft robots without incurring heavy and bulky parts such as batteries.

## 1. Introduction

Soft robots, which can move in complex environments such as curved and narrow passages, have been recently developed by mimicking the forms of worms and octopuses [1,2,3]. Soft robots have mainly been operated by using pump-generated pneumatic pressure [4,5], motor-controlled cable tendons [6,7], and electro-active polymers (EAPs) [8,9], which require a high applied voltage of several kilovolts. This implies that typical soft robots must include external heavy payloads, which limit the independent mobility of soft robots [10]. Other mechanisms, such as using shape memory alloy (SMA) or photosensitive materials, are used; however, they cannot operate under obstacles in those cut-off light [11] or with a relatively low voltage supply [12].

On the other hand, the micro thermopneumatic actuator operates with thermal expansion caused by heating the air when the electric current is supplied to the Joule heater inside the actuator [13]. The thermopneumatic mechanism is a promising alternative to the configuration of a portable system because it can replace the external power supplies needed for operating actuators with small batteries [14]. Additionally, an actuator without a battery, which is the heaviest payload for driving a heater, can be realized by introducing wireless power transfer. Wireless power transfer has been applied in several approaches: magnetic induction [15] using electromagnetic induction between transmitting and receiving inductors; magnetic coupling [16] using the coupled resonance of transmitting and receiving inductors; and microwaves [17], in which gigahertz electromagnetic waves are directly propagated through an antenna. While a complicated circuit with additional capacitors and inductors needs to match the transceiver’s resonant frequency for magnetic coupling and microwaves, a simple setup consisting of inductors for transmission and reception is sufficient for the magnetic induction technique to reduce its’ weight [18].

Moreover, because the air pressure for deformation of the thermopneumatic actuator requires a significant energy supply, actuators based on the liquid–gas phase change of substances with low boiling points have been developed [14,19]. Compared to typical thermopneumatic actuators those heat the interior enclosed air, the actuators based on the liquid–gas phase change operate with less power because these are only needed to provide power to heat a temperature above the low boiling point of the liquid. Therefore, the phase change mechanism mitigates the limitation of the thermopneumatic mechanism using wireless power transfer, which supplies a relatively small amount of electric power. Boyvat et al. presented a magnetic coupling system that delivered 0.16–1.33 W of power to a soft actuator with a size of tens of millimeters based on the liquid–gas phase change [20]. In addition, Mirvakili et al. constructed an actuator that operated by inductively heating magnetic particles mixed with deionized (DI) water and suppling power of up to 32 W for a gripping motion [21].

A micro coil, which is fabricated using microfabrication technology based on thin-film stacks, inherently has a two-dimensional spiral structure. Because the spiral inductor is located in the two-dimensional plane, it must be designed in a curved structure that rotates around a point and shrinks inward to have as many magnetic flux links as possible. Thus, when the spiral inductor is used as the receiving inductor for magnetic induction, it has a relatively lower induced voltage than a helical inductor, which has the same number of turns but repeatedly forms circles of the same diameter. Moreover, the two-dimensional heater is placed on a plane, so that the heat transfer efficiency is inevitably lower compared to the three-dimensional heater’s heat transfer efficiency. Hence, realizing a three-dimensional micro coil and utilizing all three-dimensional spaces can be a major milestone in improving the performance of wireless thermopneumatic actuators by enabling the inductor to generate greater inductive power and allowing the heater to consume less power.

In this study, a thermopneumatic soft actuator, equipped with a three-dimensional inductor that has the capability of wireless power transfer, and a three-dimensional Joule heater were developed for independent operation. The actuator was fabricated by casting elastic polymer into a mold printed using a high-precision 3D printer. It operates based on the liquid–gas phase change and is powered wirelessly by magnetic induction. The three-dimensional bellows-shaped actuator was designed through numerical analysis using COMSOL Multiphysics to increase the stroke exerted by heat. Finally, the improved wireless soft actuator of the three-dimensional helical structure was verified by comparing it with a micro two-dimensional receiving inductor with a spiral structure fabricated by conventional soft lithography.

## 2. Concept

**Figure 1:**
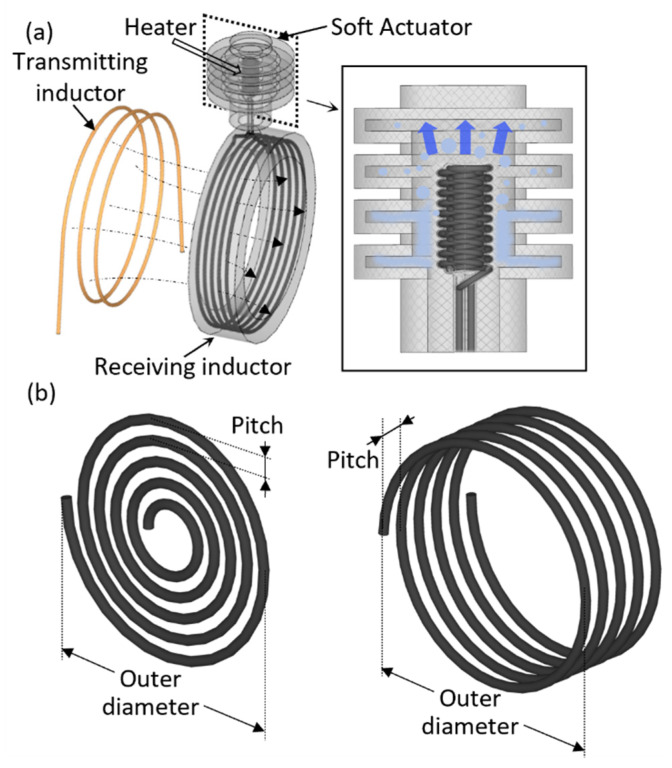
Schematics diagram of a wireless micro soft actuator: (**a**) Wireless power transfer and the following expansion of the micro soft actuator by liquid–gas phase change of working fluid inside the actuator. (**b**) Multi-turn coils with two-dimensional spiral structure (**left**) and three-dimensional helical structure (**right**).

We devised a soft bellows-shaped actuator that has independent action by employing three-dimensional micro inductors, instead of using conventional planar micro inductors to improve wireless power supply by magnetic induction. The magnetic induction is a method in which the wireless power transfer is unaffected by variations in the transmitting and receiving inductors that may be caused by the motion of the actuator and the resulting change in the resonant frequency. Moreover, a thermopneumatic soft actuator was realized by adopting a three-dimensional micro Joule heater to improve the heat transfer and by using working fluid for liquid–gas phase changes to maximize the exerted stroke using the supplied power. As shown in Figure 1, a time-varying magnetic field is formed as AC power which flows in the transmitting inductor, simultaneously resulting in electromagnetic induction in the receiving inductor, which induces the voltage. By the voltage generated from the receiving inductor, the Joule heater heats and evaporates the liquid filled in the actuator, ultimately increasing the inner pressure to expand the actuator. While the voltage applied to the transmit inductor is cut off, the voltage in the receiving inductor disappears, and the heater stops heating. The evaporated working fluid, therefore, loses heat on account of ambient temperature and returns to the liquid phase. This reduces the internal pressure of the actuator and returns it to its initial state by contraction.

**Figure 2:**
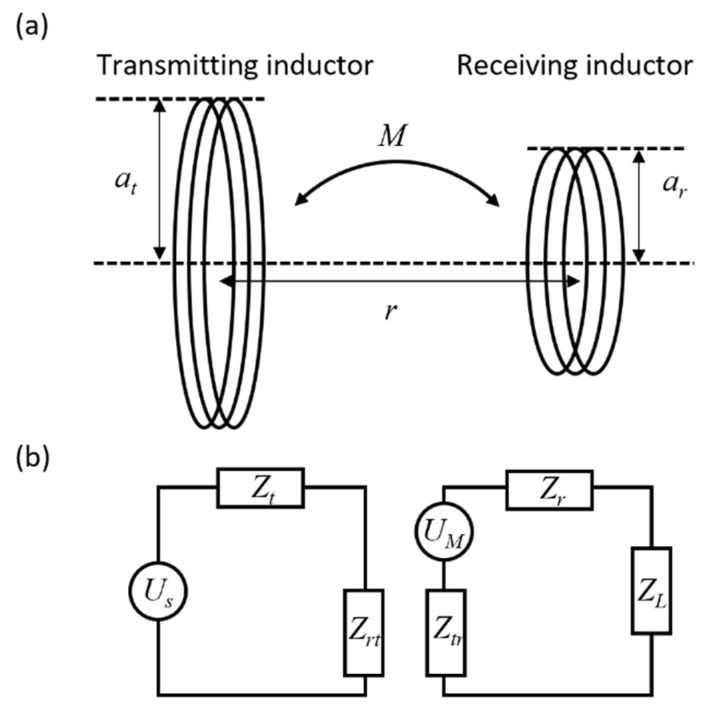
Wireless power transfer based on magnetic induction: (**a**) Magnetically coupled transmitting and receiving inductor. (**b**) Equivalent circuit containing source, mutually coupled inductors, and load (i.e., Joule heater).

In previous studies, using wireless power transfer micro receiving inductors had a fabrication process such as depositing and etching thin metal films. To increase an induced voltage of the receiving inductor, imposed by the standard microfabrication within the two-dimensional structural limit, the receiving inductor has generally been fabricated as a spiral structure that diverges from a point, as shown in Figure 1b. Therefore, as the spiral inductor’s number of turns increases, the area surrounded by each turn decreases proportionally. Here, as shown in Figure 2a, when the radii of each inductor are *a_t_* and *a_r_*, and the distance between two centers is *r*, the mutual inductance *M* between the two inductors can be calculated as follows [22]:

(1) $$M=\frac{N_{r}\oint_{l_{r}}A\cdot d{\vec{l}}_{r}}{dI}≃\mu \pi N_{t}N_{r}\frac{a_{t}^{2}a_{r}^{2}}{2r^{3}}$$

As shown in Figure 2b, for the equivalent circuit, induced voltage *U_M_* is expressed as follows:

(2) $$U_{M}=-j\omega M\frac{\;U_{s}}{\;R_{t}+j\omega L_{t}}≃-\mu \pi N_{t}N_{r}\frac{a_{t}^{2}a_{r}^{2}}{2r^{3}}\cdot \frac{\;U_{s}}{\;R_{t}+j\omega L_{t}}$$

In a closed circuit, the same current flows through *Z_r_*, which is the self-impedance of the receiving inductor, and *Z_L_*, which is the load impedance connected to the receiving inductor. In addition, it flows through *Z_tr_*, which is the mutual impedance due to the magnetic coupling between the two inductors. Thus, voltage *U_L_* across *Z_L_* is calculated by the voltage division as follows:

(3) $$U_{L}=\frac{Z_{L}}{Z_{r}+Z_{L}+Z_{tr}}U_{M}$$

Voltage *U_L_* applied to the heater is proportional to the area of the receiving inductor. Therefore, the induced voltage of the helical inductor—having a constant cross-sectional area enclosed by the circles of each turn engendered by the three-dimensional structure—is greater than that of the two-dimensional spiral inductor.

**Figure 3:**
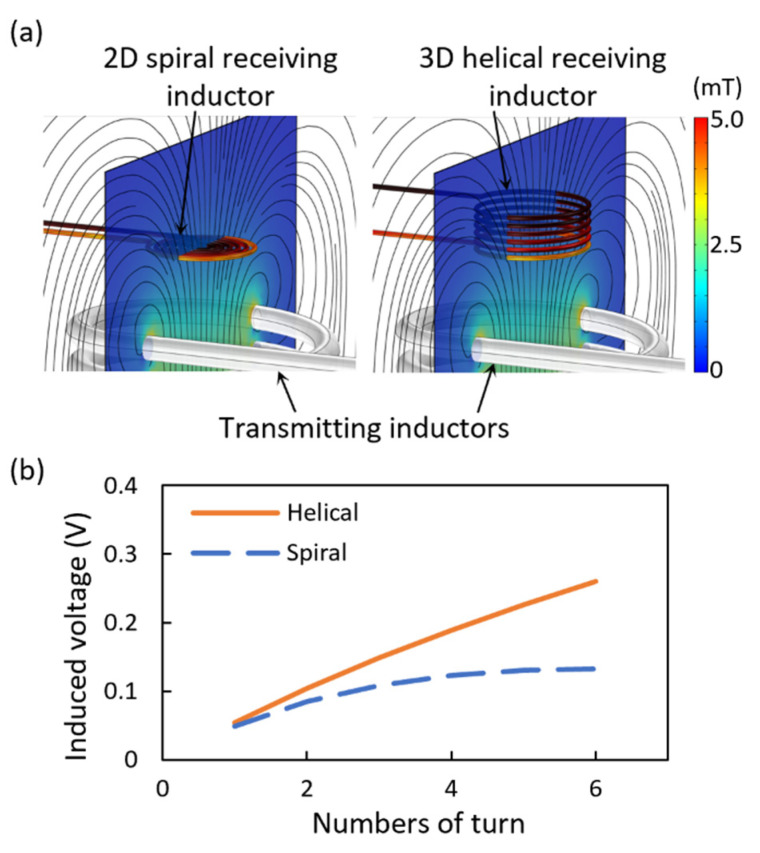
Comparison between a two-dimensional spiral and a three-dimensional helical receiving inductor: (**a**) The magnetic flux density around the inductors is displayed on the surface containing the center of the inductors, and the pattern of the magnetic field is shown as the streamline. (**b**) Induced voltages according to the number of coil turns in the two-dimensional spiral and the three-dimensional helical inductor.

As shown in Figure 3, the magnetic induction for the receiving inductors of the two-dimensional spiral structure and the three-dimensional helical structure is numerically calculated using the magnetic field module of COMSOL Multiphysics^®^ (Stockholm, Sweden). Figure 3a shows the magnetic flux density induced around the inductors using an identical transmitting inductor. The transmitting inductor has an outer diameter of 20 mm and has ten turns, where a 192 kHz voltage with the maximum amplitude of 11.4 V is applied. The strength of the magnetic flux density was compared according to the number of coil turns of the receiving inductors with the two-dimensional spiral and the three-dimensional helical structure, those that have identical coil thickness, outer diameter, and pitch. As shown in Figure 3b, as the number of turn increase, the difference between the induced voltage of the helical and spiral receiving inductors gradually increases. This is because, the area of the spiral inductor decreases while the coil’s number of turns increases, but the area of the helical inductor does not change. Therefore, it was confirmed that connecting a three-dimensional helical inductor instead of the two-dimensional plane spiral inductor increases the induced voltage and the transmission distance.

## 3. Materials and Methods

### 3.1. Fabrication

**Figure 4:**
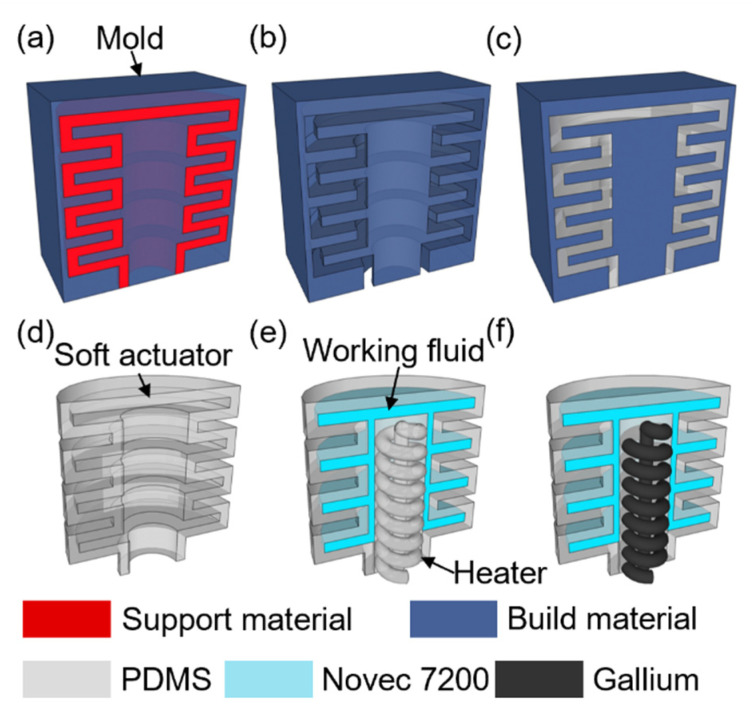
The fabrication process of the three-dimensional micro bellows actuator for the liquid–gas phase change mechanism: (**a**) The mold of the actuator is printed with the build and the support material. (**b**) The support material filled inside the mold is selectively removed by a dewaxing solvent. (**c**) Liquid PDMS is poured into the mold and cured in a convection oven. (**d**) The mold made of build material is dissolved with acetone, leaving the cast bellows actuator. (**e**) Working fluid and PDMS heater are sequentially inserted into the bellows actuator. (**f**) Liquid gallium is injected with a syringe into the channel of the PDMS heater to enable electrical conductivity. When the heater is heated due to the induced electromotive force by the receiving inductor, the working fluid inside the actuator evaporates and the internal pressure increases. The increased pressure causes the PDMS actuator to expand.

The micro soft bellows-shaped actuators’ soluble mold was designed by using 3D CAD (Inventor, Autodesk, Mill Valley, CA, USA). The soluble mold was printed with a 3D printer (3Z STUDIO, Solidscape, Merrimack, NH, USA), and to provide supporting structures for the build, the empty space inside the mold was filled with the support material (Figure 4a). The support material was selectively dissolved by steeping the mold in a 50 °C dewaxing solvent (BIOACT^®^ VSO, Petroferm, Gurnee, IL, USA) stirred at 400 rpm for 6 h (Figure 4b). A polydimethylsiloxane (PDMS) (Sylgard 184, Dow Corning, Midland, MI, USA) mixture with a mass ratio of 15:1 was poured into the mold, and the bubble was removed in a vacuum chamber so that the liquid PDMS could be filled in the mold (Figure 4c). To chemically remove the mold after casting the PDMS, the mold with the casted structure inside was dissolved in acetone for 30 min, followed by cleaning with DI water (Figure 4d). Using the same process, three-dimensional PDMS channels for the heater, and receiving inductor for injecting liquid metal were prepared. For the liquid–gas phase change, the empty space inside the actuator was filled with working fluid (Novec 7200) by evacuating the air bubbles inside the bellows-shaped actuator submerged in the working fluid by vacuum. Meanwhile, the PDMS channel is inserted for the Joule heater while pushing out some of the working fluid inside the actuator, and then the liquid PDMS mixture was covered and cured inside the gap between the actuator and the channel (Figure 4e). Finally, the empty channel for the heater is injected with liquid gallium using a syringe, and the heater and receiving inductor were electrically connected through copper wires. The filler hole was sealed by pasting and curing liquid PDMS (Figure 4f).

### 3.2. Experimental Setup

**Figure 5:**
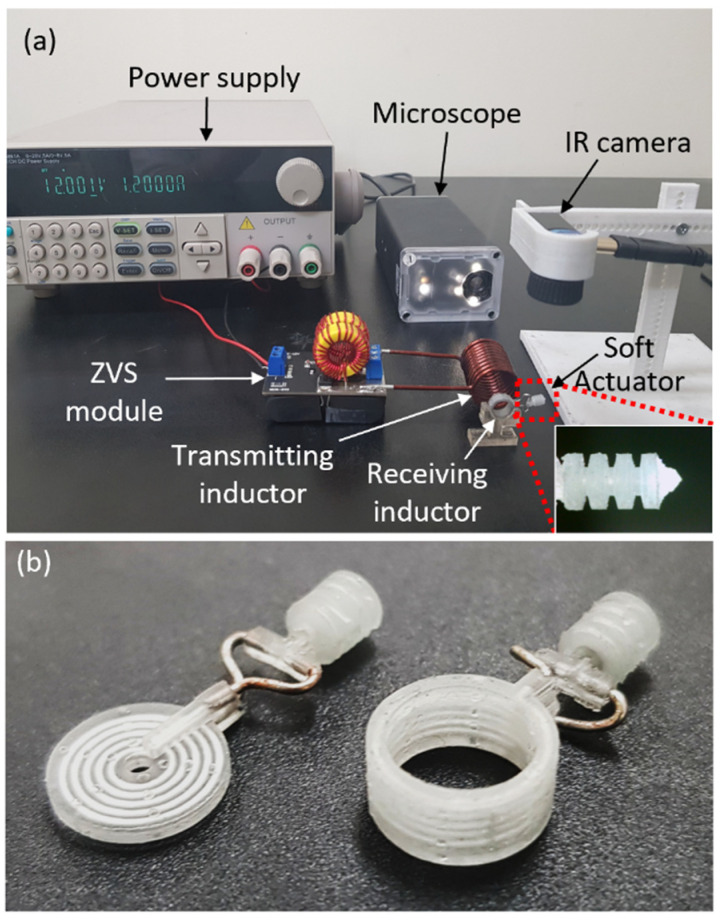
Measurement setups for the wirelessly powered bellows actuator characterization: (**a**) Experimental configuration measuring the operation of the soft bellows-shaped actuator with an optical microscope. (**b**) The soft bellows-shaped actuator has a built-in heater that is connected to the receiving inductor by wires.

As shown in Figure 5a, an experiment was set up to measure the displacement of a micro soft bellows-shaped actuator by magnetic induction. The transmitting inductor is connected to a zero-voltage switching (ZVS) module that operates with a 12 V DC received from a power supply. The time-varying magnetic field was generated by the ZVS module as it applies a voltage of 192 kHz with 11.4 V maximum amplitude to the transmitting inductor. Through electromagnetic induction, the time-varying magnetic field transmits power to the receiving inductor. The soft bellows actuator expands when the Joule heater generates the heat by the voltage induced in the receiving inductor. As the induced voltage in the receiving inductor supplies power to the Joule heater, the Joule heater heats the internal working fluid and consequently results in micro soft bellows-shaped actuator’s deformation.

The temperature distribution was measured by using an infrared camera (Seek Thermal Pro, Seek Thermal, Santa Barbara, CA, USA), and the stroke of the bellows-shaped actuator was measured over time using an optical microscope (UM12, Microlinks Technology, Kaohsiung, Taiwan). It was possible to adjust the transmission distance and misalignment distance between the two inductors using a custom-made stage. Figure 5b shows the entire system of the soft bellows-shaped actuator in which the receiving inductor and the heater made with the liquid metal are combined with each other. The performance of the receiving inductors with two-dimensional spiral and three-dimensional helical structures is evaluated through the experiment in Section 4.1.

## 4. Results and Discussion

### 4.1. Characterization of Heater and Receiving Inductor

To heat above Novec 7200’s boiling point, which gets inside the micro soft bellows-shaped actuator, the Joule heater must have sufficient heating capacity. The heater made with liquid metal is designed to be centered, considering the bellows-shaped actuator’s size, and its measured resistance at room temperature is 164 mΩ. Spiral inductor and helical inductor are manufactured using the same process to compare their characteristics and the resistance of both inductors was measured by using an LCR meter. The resistance measured is listed in Table 1.

**Figure 6:**
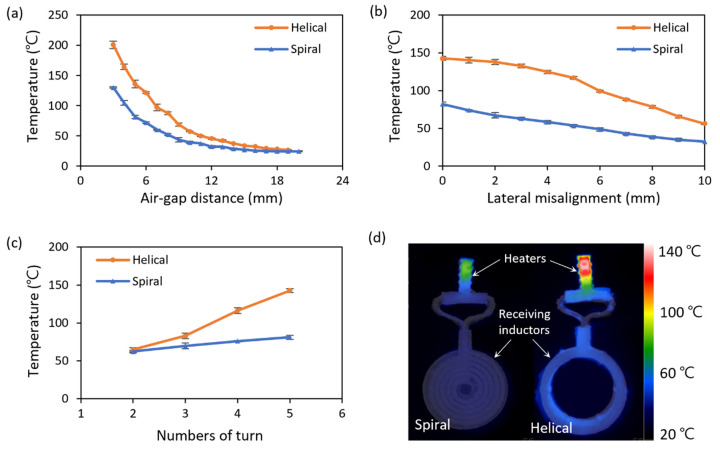
Characteristics of the heater connected to the two-dimensional spiral and the three-dimensional helical inductor: Temperature change of the heaters (**a**) according to the air-gap distance between the transmitting inductor and the receiving inductor, (**b**) along the misaligned distance, and (**c**) according to winding numbers of the heaters. (**d**) Temperature distribution of the heaters and the receiving inductors, captured by an infrared camera.

In Figure 6, the temperature of the heaters generated by the power, transferred from the receiving inductors with the two-dimensional spiral and the three-dimensional helical structure, is compared according to the location of the receiving inductor. Figure 6a shows the temperature of the heater, which varies according to the air-gap distance between the coil when the center axis of the transmitting inductor and the receiving inductor is matched. The two-dimensional spiral inductor heated the Joule heater up to 81.3 °C when the air-gap distance was 5 mm, while the three-dimensional helical inductor heated the Joule heater up to 136.0 °C when the air-gap distance was 5 mm. Meanwhile, to increase the heater temperature to 76 °C, which is the boiling point of Novec 7200, the helical inductor could be separated from the transmitting inductor by 154.5% farther than the spiral inductor.

Figure 6b shows the change in heater temperature due to lateral misalignment, which is the parallel distance between the two-center axis of the transmitting and receiving inductors. The air-gap distance between the transmitting and receiving inductors is 5 mm, which is the same for both coils. When the receiving inductor is misaligned from the axis of the transmitting inductor, the transmitted power is reduced owing to the decrease in the magnetic flux linkage. The temperatures of the heater connected to the spiral inductor and helical inductor decrease by 60.5% and 60.7%, respectively, when the receiving coils are misaligned by 10 mm. The reason why the decreasing rates of the two are similar in that the magnetic flux linkage is directly proportional to the area where the cross-sections of the transmitting inductor and the receiving inductor overlap.

In Figure 6c, the power transfer is conducted while the center axis of each coil is aligned, and the air-gap distance is 5 mm. When the coil’s number of turns is two, the temperature of the heater attached to the three-dimensional helical inductor increases by 2.3 °C (103.7%) more than that with the two-dimensional spiral inductor; however, when the coil’s number of the turn is five, it is observed to increase by 61.7 °C (176.1%). This is because as the number of turns increases, the magnetic flux linkage increases, owing to the structural differences of the coils.

Figure 6d represents the infrared image of the receiving inductors and heaters with an air-gap distance of 5 mm. As the channel diameter of the receiving inductor is thicker than that of the heater, the temperature rise of the receiving inductor is not significant, so electrical disconnection does not occur, and a stable power supply is possible.

We operated the heater temperature to below 200 °C. This is because the heater consists of gallium, which is a liquid metal with high electrical conductivity, and PDMS was employed to maintain its surrounding shape. This means that the thermal expansion coefficient of PDMS is approximately eight times higher than that of gallium, resulting in an empty space inside the heater as the heat increases [23]. In Figure S1, we measured the resistance of the heater by applying the current of 1.2–2.8 A to the heater to consider the electric circuit opening that occurred when the actuator is operated. Moreover, the surrounding pressure of 0 and 10 kPa was applied to the heater, considering the pressure applied to the heater inside the actuator. According to the experimental results, the electric circuit was opened when a current of greater than 2 A is applied to the heater at 10 kPa. Therefore, the heater needs to operate at less than 2 A for repeated actuating at 10 kPa where the actuator is fully expanded without damage.

### 4.2. Characterization of Wireless Bellows-Shaped Actuator

**Figure 7:**
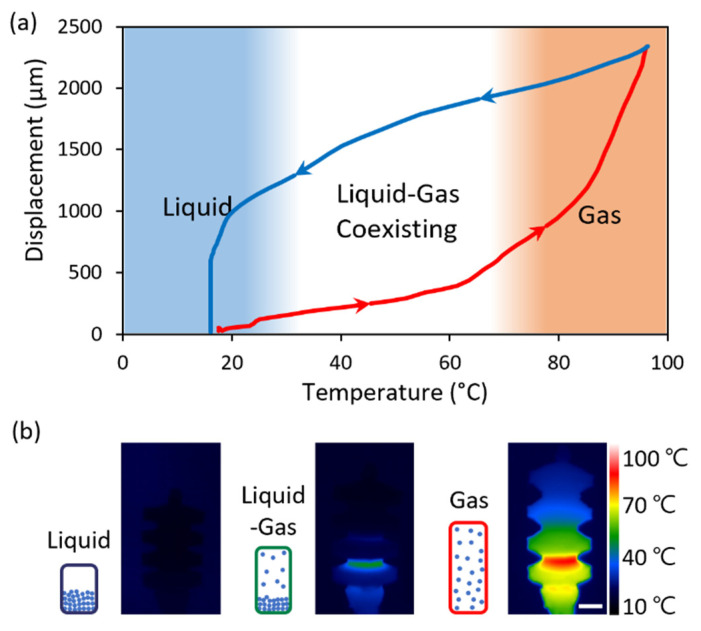
Response of the bellows actuator by the liquid–gas phase change: (**a**) Displacement according to the bellows surface temperature. The displacement increases along the red curve during heating and decreases along the blue curve during cooling owing to the powering off. (**b**) IR images of the actuator at each liquid–gas phase stage. The scale bar indicates 1 mm.

As shown in Figure 7, the feature of the stationary temperature of the bellows-shaped actuator depending on the heater was investigated. The temperature in Figure 7b is the surface temperature measured by using an infrared camera, with consideration of the heater position and the difference in the temperature inside the actuator due to the low thermal conductivity factor (~0.15 W m^−1^ K^−1^).

As shown in Figure 7a, the state of the working fluid inside the actuator is divided into three stages [24]. The temperature range of each stage was determined by optically confirming the boiling of Novec 7200 inside the semi-transparent PDMS actuator. In the first stage, where the temperature is lower than the boiling point of the working fluid, the effect of the generated heat on the displacement is insufficient because it is used to raise the temperature of the liquid. In the second stage, when the temperature begins to rise above the boiling point of the working fluid, the generated heat is used for the phase change of the working fluid, and the inner pressure of the actuator is increased by the gas. Thus, the liquid and gas coexist, and the actuator begins to move owing to the pressure of the evaporated gas. Finally, in the third stage, all the liquid inside the actuator exists in the gas phase, creating a large strain in the elastomer. Additionally, during cooling, the temperature of the PDMS surface is quickly cooled by natural convection; however, owing to the low thermal conductivity of PDMS, the temperature inside the actuator decreases relatively slowly, which is represented as the hysteresis of the displacement. In Figure 7b, the displacement and surface temperature distribution of the actuators are exhibited using the infrared camera when the temperatures of the actuators are 20, 50, and 80 °C, respectively.

**Figure 8:**
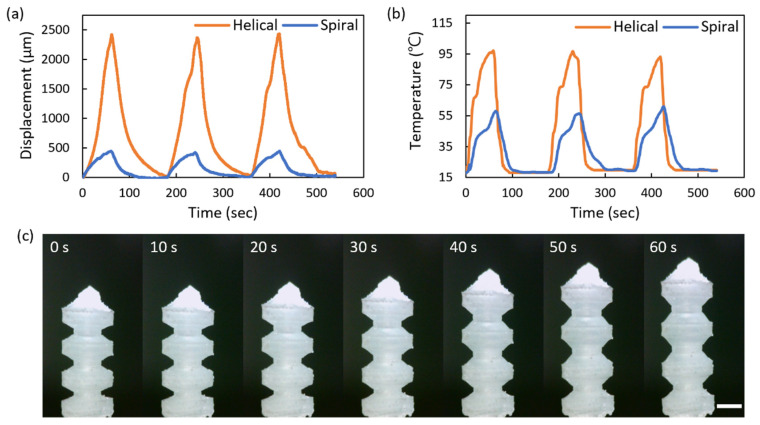
Performance of the thermopneumatic bellows actuator connected to the two-dimensional spiral and the three-dimensional helical inductor: (**a**) Real-time displacement of the actuators. (**b**) Temperature change of the outer surface of the actuators. (**c**) The captured driving actuator connected to the three-dimensional helical inductor. The scale bar indicates 1 mm.

Figure 8 and Supplementary Video S1 illustrate the micro soft bellows-shaped actuator’s performance while the power supply is repeatedly switched on for 60 s and then off for 120 s. Figure 8a indicates the displacement of the bellows-shaped actuator connected to the spiral and the helical inductor, respectively, while the distance of air-gap is 5 mm with the coincided center axis of the transmitting and receiving inductors. When connected to the helical inductor, the bellows-shaped actuator exerts up to 2427 μm of displacement, which is 522% greater than that when connected to the spiral inductor. The helical inductor has higher temperature heaters because it links more magnetic flux than the spiral inductors, which enables longer strokes by causing more liquid evaporation. In Figure 8b, the temperature change of the bellows-shaped actuator is measured over time. Compared to the spiral inductor, the helical inductor increases the displacement of the actuator up to 5.22 times longer even with a temperature difference of 1.76 times. This is because the working fluid in the actuator with the spiral inductor is only heated up to the liquid–gas coexisting stage, while the working fluid in the actuator with the helical inductor is heated up to the gas stage where the entire liquid evaporates. Figure 8c shows the images observed at intervals of 10 s. Even though the displacement of 2427 μm was reached, the bellows-shaped actuator was driven below 97 °C and was therefore considered useful for temperature-sensitive conditions.

## 5. Conclusions

By using a soluble cast mold based on 3D printing technology and by using biocompatible PDMS and liquid metal, a 3D helical inductor and a 3D helical Joule heater, which were unattainable to produce with conventional microfabrication, were designed and realized. Moreover, an untethered soft micro bellows-shaped actuator was designed, manufactured, and evaluated, by using the power, which was supplied by the magnetic induction, and by using the liquid–gas phase change. To this end, the developed 3D helical receiving inductor and the 3D helical Joule heater were utilized. First, the 3D helical inductor increased the temperature of the connected 3D Joule heater by up to 176% compared to the conventional 2D spiral inductor, resulting in a 522% increase in the stroke of the bellows actuator. Second, the magnetic induction-based wireless power transfer enabled to implement a system without the battery and drive circuit, which are large and heavy payloads compared to a single light inductor, and also supplied sufficient power to generate the liquid–gas phase change to drive the actuator. Finally, by using the working fluid with a boiling point of 76 °C, the temperature of the actuator with a maximum displacement of 2427 μm was maintained below 97 °C, showing the potential for various applications. Ultimately, the micro actuator with 3D helical inductors, which improved the efficiency of the wireless power transmission, demonstrated the possibility to develop independent soft micro-robots those operate in environments without heavy payloads.

## Figures and Tables

**Table 1:** Key dimensions and measured parameters of transmitting and receiving inductors.

	Coil Thickness [mm]	Outer Diameter [mm]	Pitch [mm]	Turns	Resistance [mΩ]	Inductance [μH]
Transmitting inductor	2	20	1	10	3.8	1.32
2D spiral receiving inductor	0.4	9	1	5	244	0.46
3D helical receiving inductor	0.4	9	1	5	321	0.58

## Data Availability

The data presented in this study are available within the article. Other data that support the findings of this study are available upon request from the corresponding author.

## Supplementary Materials

The following supporting information can be downloaded at: https://www.mdpi.com/article/10.3390/mi13050799/s1, Figure S1: The resistance of the heater according to the adjustment of the external air pressure when applied the current, Video S1: repeated operation of the thermopneumatic bellows actuator.
