# Supplementary material for: Wireless Micro Soft Actuator without Payloads Using 3D Helical Coils
Source: Micromachines (Basel). 2022 May 20;13(5):799. doi: 10.3390/mi13050799 (PMC9143378; doi:10.3390/mi13050799)
Supplement: Supplementary file 1 [file micromachines-13-00799-s001.zip › supplementary .pdf]

Wireless Micro Soft Actuator without Payloads Using 3D Helical Coils

Seonghyeon Lee, Woojun Jung, Kyungho Ko, and Yongha Hwang*

Department of Control and Instrumentation Engineering, Korea University, Sejong, Republic of Korea; kazamajin95@korea.ac.kr (S.L.); wjkst2010@korea.ac.kr (W.J.); gokyungho123@korea.ac.kr (K.K.)

* Correspondence: hwangyongha@korea.ac.kr

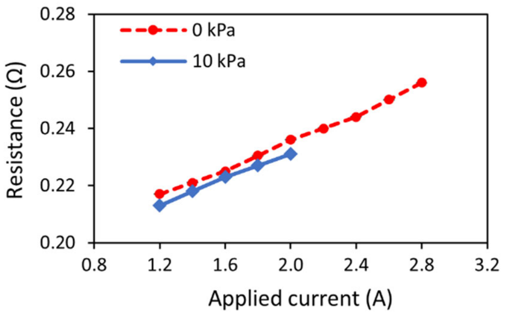

Figure S1. The resistance of the heater according to the adjustment of the external air pressure of 0 and 10 kPa when applied the current of 1.2 – 2.8 A to the heater. The electric circuit of the heater is opened when a current of greater than 2 A is applied to the heater at 10 kPa.
